# Neoadjuvant Therapy for Non-melanoma Skin Cancer: Updated Therapeutic Approaches for Basal, Squamous, and Merkel Cell Carcinoma

**DOI:** 10.1007/s11864-021-00826-3

**Published:** 2021-03-16

**Authors:** Enrico Zelin, Iris Zalaudek, Marina Agozzino, Caterina Dianzani, Arianna Dri, Nicola Di Meo, Roberta Giuffrida, Giovanni Francesco Marangi, Nicoleta Neagu, Paolo Persichetti, Ludovica Toffoli, Claudio Conforti

**Affiliations:** 1 Dermatology Clinic, Maggiore Hospital, University of Trieste, Piazza dell’Ospitale 1, 34129 Trieste, Italy; 2 Plastic and Reconstructive Surgery Department, Campus Biomedico University, Rome, Italy; 3 Department of Clinical and Experimental Medicine, Dermatology Section, University of Messina, Messina, Italy; 4 Dermatology Clinic, Mures Country Hospital, Tirgu Mures, Romania

**Keywords:** Non-melanoma skin cancer, Basal cell carcinoma, Squamous cell carcinoma, Merkel cell carcinoma, Neoadjuvant treatment, Targeted therapy, Immunotherapy, Vismodegib, Sonidegib

## Abstract

Recently introduced systemic therapies for locally advanced and metastatic non-melanoma skin cancers (NMSCs) are paving the way for neoadjuvant approach. Although none of the therapeutic options has currently gained indication in this setting, neoadjuvant approach for NMSCs is an open field and we are likely to see huge developments in the near future. Targeted therapy with sonic hedgehog pathway inhibitors is very effective in locally advanced or multiple basal cell carcinomas while immunotherapy with immune checkpoint inhibitors appears to be promising for advanced cutaneous squamous cell carcinoma and Merkel cell carcinoma. To date, targeted therapy and immunotherapy represent the frontiers in NMSC therapeutic management and, according to recent studies, good results can be achieved.

## Introduction

### Non-melanoma skin cancers: background

Non-melanoma skin cancers (NMSCs) are the most common malignancies, accounting for up to 30% of all human tumors [1, 2]. Basal cell carcinomas (BCCs) and cutaneous squamous cell carcinomas (cSCCs) are keratinocyte cancers and represent the most common NMSCs, while rarer tumors included in this category are Merkel cell carcinoma (MCC), cutaneous sarcomas, appendageal tumors, and cutaneous lymphomas [2]. The incidence of keratinocyte cancers is steadily growing, probably due to different factors (ageing population, increasing ultraviolet radiation exposure, raising number of immunosuppressed individuals, and possibly greater awareness of the disease and earlier diagnosis) [1, 3]. BCC to cSCC incidence ratio varies between 4:1 and 1:1, according to different sources [1–3]. Although the primary aim is to perform an early diagnosis through minimally invasive procedures such as dermoscopy [4–6], advanced tumors are not uncommon and require a more challenging treatment.

### Conventional treatment options and definition of neoadjuvant approach

Surgical excision is regarded as the therapeutic gold standard for most NMSCs and together with radiotherapy is considered a potentially curative treatment [7]. Radiotherapy is an effective non-surgical option and it is used in the definitive, adjuvant, and palliative settings for these malignancies [7, 8•]. It is well-tolerated and may offer a better cosmetic and functional outcome in comparison with surgery [7, 8•]. NMSCs are radioresponsive and local control rates are excellent, irrespective of the radiotherapy dose or dose per fraction (local control rate at 5 years of 92% for cSCC and 96% for BCC, according to a large study involving 597 patients with 1021 lesions) [9].

In some cases, surgery or radiotherapy cannot be adopted as first step; therefore, a neoadjuvant treatment is indicated. By definition, a neoadjuvant approach aims to reduce the size of the tumor, in order to make it eligible for other potentially curative techniques. The most typical circumstance requiring a neoadjuvant treatment regards advanced tumors, when there may be a significant risk of functional and cosmetic deficit from immediate surgery because of size, number, or location of the cancers [10, 11]. In evaluating tumor response to treatment, RECIST (Response Evaluation Criteria In Solid Tumors) criteria are often used, defining complete response (CR), partial response (PR), stable disease (SD), and progressive disease (PD) [12, 13].

Herein, we report the most commonly pharmacologic treatments and interventional procedures in advanced NMSC. Currently, none of them has gained indication as neoadjuvant therapy, and evidence on their efficacy in this setting is limited and based on case reports, case series, and often non-randomized small studies. Notably, there are no reports of radiotherapy used as neoadjuvant option before surgery in NMSCs, because irradiated tissues are deemed to present poor wound healing [3].

## Pharmacologic treatments: targeted therapy

Targeted therapy interferes with cancer cell growth by blocking specific target molecules needed for carcinogenesis and tumor development. It is much more specific than traditional chemotherapy, which simply affects all rapidly dividing cells. However, targeted therapy is clearly not simple to develop, since these target molecules have to be precisely identified; subsequently, a specific drug that blocks them has to be engineered and the clinical benefit has to be ultimately demonstrated. Vismodegib and sonidegib are sonic hedgehog pathway inhibitors approved for locally advanced and/or metastatic BCCs [14••]. There are no targeted therapies currently approved for advanced cSCC but epidermal growth factor inhibitors have been used so far in some cases [15–21].

### Sonic hedgehog pathway inhibitors

The sonic hedgehog pathway is involved in cell differentiation and proliferation and its dysregulation is very important in BCC genesis [2, 14••]. Patch 1 (encoded by *PTCH1* gene) is a transmembrane protein that acts as tumor suppressor by inhibiting smoothened (SMO), another transmembrane protein. Physiologic ligands (e.g., sonic hedgehog itself) binding to PTCH1 or loss-of-function PTCH1 mutations (frequently found in both sporadic BCCs and Gorlin-Goltz syndrome-associated BCCs) relieve this inhibition of SMO. This ultimately leads to nuclear translocation of GLI transcription factors and activation of target genes, causing cell proliferation and tumor growth [2]. Vismodegib and sonidegib act by antagonizing SMO and are both currently licensed for treatment of locally advanced basal cell carcinomas (la-BCCs) [14••]. Vismodegib is also approved for metastatic basal cell carcinomas (m-BCCs) [14••].

#### Vismodegib

Efficacy and safety of vismodegib were firstly assessed in 104 patients suffering from la-BCC (*n*=71) and m-BCC (*n*=33) during a follow-up of 39 months in the phase II non-randomized clinical trial ERIVANCE [22, 23]. Response rate (RR, percentage of complete or partial responses) by investigator review was 60.3% for la-BCC and 48.5% for m-BCC, with a median response duration of 26.2 and 14.8 months, respectively [24]. Of note, independent review facility-assessed RR was 43% in laBCC and 30% in mBCC [24]. Similar results were reported by the STEVIE trial (based on 1215 patients, 1119 with la-BCC and 96 with m-BCC): investigator-assessed RR was 68.5% for la-BCC and 36.9% for m-BCC [25, 26].

Vismodegib was also used in combination with radiotherapy in la-BCC with good control of disease, according to a case series and a study involving 24 patients (NCT01835626) [27].

In consideration of its efficacy in tumor burden reduction, vismodegib could be very promising as neoadjuvant treatment in real-life setting, above all in la-BCCs, although strong data and evidence are lacking. In 15 patients with la-BCC treated with neoadjuvant vismodegib and subsequent surgical excision, after a follow-up of almost 2 years, only one recurrence was reported [28]. Moreover, there are other ongoing studies investigating the potential of this drug in neoadjuvant setting (NCT03035188, NCT02667574).

##### Standard dosage

150 mg/day, oral administration

##### Contraindications

Hypersensitivity to active principle or bulking agents, pregnancy and lactation, inadequate pregnancy prevention.

##### Main drug interactions

When administered together with drugs that increase gastric pH, vismodegib absorption can be reduced. The concomitant use of CYP3A4 inductors (such as rifampicin, carbamazepine, phenytoin, and St. John’s herb or *Hypericum perforatum*) may reduce exposition to vismodegib, while contemporary administration of CYP3A4 inhibitors (e.g., azoles) or P-glycoprotein inhibitors (e.g., clarithromycin, erythromycin, azithromycin) may increase it, but these latter interactions are not clinically significant.

Vismodegib may decrease the efficacy of steroid contraceptives and increase the exposition to drugs transported by breast cancer resistance protein (BCRP) (e.g., rosuvastatin, topotecan, sulfasalazine) and to OATP1B1 substrates (e.g., statins, ezetimibe, valsartan, and some antidiabetic drugs) [29, 30].

##### Main adverse effects

Muscular spasms (66%), alopecia (62%), dysgeusia (55%), weight loss (41%), decreased appetite (25%), asthenia (24%), nausea (17.9%), ageusia (17.5%), fatigue (16.5%), diarrhea (16.2%), arthralgia (10.2%) [26]. Other adverse effects (reported in <10% of patients) are headache, anemia, abdominal pain, cough, pruritus, skin rash, increased levels of liver enzymes, creatine phosphokinase and creatinine, and increased risk of cSCC [26].

##### Special points

Among sonic hedgehog pathway inhibitors, vismodegib is the only one approved for m-BCC.

##### Cost/cost-effectiveness

The cost is high, considering that this therapy should be maintained as long as possible.

#### Sonidegib

Long-term efficacy and safety of sonidegib were assessed during the randomized phase II trial BOLT, recruiting 230 patients with la-BCC (*n*=194) or m-BCC (*n*=36) divided in two arms according to a 2:1 ratio (the first arm receiving 800 mg/day of sonidegib and the second 200 mg/day, respectively) [31]. The dose of 200 mg/day was the only one to obtain approval, because it was effective and better tolerated [31]. After a median follow-up of 30 months, investigator-assessed RR was 71.2% for la-BCC and 23.1% for m-BCC, with median response duration of 15.7 and 18.1 months, and 2-year survival rates of 93.2 and 69.3%, respectively [31]**.** RR assessed per central review was 56.1% for la-BCC and 7.7% for m-BCC [31]**.** Notably, in BOLT trial, tumor response for la-BCC was evaluated using BCC-modified Response Evaluation Criteria In Solid Tumors (mRECIST), which are more stringent than those used in ERVANCE [31]**.**

There is an ongoing study evaluating sonidegib in neoadjuvant setting for la-BCC, followed by surgery or imiquimod (NCT03534947).

##### Standard dosage

200 mg/day or 200 mg every other day, oral administration.

##### Contraindications

Hypersensitivity to active principle or bulking agents, pregnancy and lactation, inadequate pregnancy prevention.

##### Main drug interactions

When administered together with drugs that increase gastric pH, sonidegib absorption may be reduced. Since sonidegib is primarily metabolized by CYP3A4, its inhibitors (e.g., ritonavir, saquinavir, telithromycin, ketoconazole, itraconazole, voriconazole, posaconazole, nefazodone) or inductors (e. g. rifampicin, rifabutin carbamazepine, phenytoin, phenobarbital, and St. John’s herb or *Hypericum perforatum*) can increase or reduce sonidegib concentration, respectively. Moreover, sonidegib is an in vitro inhibitor of CYP2B6, CYP2C9, and BCRP [32].

##### Main adverse effects

Muscular spasms (54%), alopecia (49%), dysgeusia (44%), nausea (39%), diarrhea (32%), weight loss (30%), increased levels of creatine phosphokinase (30%), fatigue (30%), decreased appetite (23%), myalgia (19%), and vomiting (11%). Other registered adverse effects were abdominal pain, anemia, headache, pruritus, increased levels of liver enzymes, lipase, amylase and creatinine, increased risk of cSCC [31]. However, the possibility to use the dosage of 200 mg every other day improves patient compliance, reducing the side effects [33].

##### Cost/cost-effectiveness

The cost is high, considering that this therapy should be maintained as long as possible.

### Epidermal growth factor receptor inhibitors

The epidermal growth factor receptor (EGFR) signaling pathway plays an important role in keratinocyte proliferation and survival and seems to be involved in cSCC carcinogenesis [34••]. EGFR inhibitors include monoclonal antibodies that bind to the extracellular domain of EGFR (e.g., cetuximab, panitumumab) but also tyrosine kinase inhibitors (TKIs), small molecules that block the activity of the EGFR tyrosine kinase domain, therefore inactivating intracellular signaling (e.g., erlotinib, gefitinib, lapatinib, dacomitinib). It is essential to underline that none of these drugs is approved for cSCC but they have been sometimes used off-label. Mutations of EGFR or molecules involved in the downstream signaling pathway (such as RAS and BRAF) can lead to resistance to EGFR inhibitors, but this occurrence is very uncommon for cSCC [21, 34••].

#### EGFR monoclonal antibody inhibitors

Cetuximab (human-murine chimeric monoclonal IgG1 antibody) is approved in colorectal cancer and head and neck SCC but has been used alone or in combination with either radiotherapy or platinum-based chemotherapy for locally advanced cSCC (la-cSCC) and metastatic cSCC (m-cSCC) [21, 35, 36]. Panitumumab (fully humanized monoclonal IgG2 antibody) is approved for colorectal cancer and has also been used in cSCC [16].

#### Cetuximab

In a phase II open-label prospective trial involving 36 patients, cetuximab was used as first-line treatment for unresectable cSCC [15]. The best overall RR was 28%, disease control rate (DCR, percentage of patients who achieved complete response, partial response, or stable disease) at 6 weeks was 69%, but the median progression-free survival (PFS) was only 4.1 months (95% CI, 1.7 to 5 months) [15]. Similar data were reported in a retrospective case series involving 31 patients with advanced cSCC, with RR of 48.5%, DCR at 6 weeks of 67.8%, and median PFS of 9 months (range, 0–36 months) [21].

Cetuximab combination with radiotherapy or platinum-based chemotherapy allows to reach a greater DCR but response remains short-lived [35, 37, 38]. As a matter of fact, in a phase II open-label prospective trial involving 20 patients with cSCC deemed as inoperable, after 2 months of treatment, RR and PFS were 80% and 1.6 months for cetuximab plus radiotherapy, 37.5% and 2.8 months for cetuximab plus carboplatin, 33% and 1.3 months for cetuximab monotherapy, respectively [37]. In a retrospective study including 12 patients with la-SCC treated with both cetuximab and radiotherapy, RR was 64% and median PFS was 6.4 months [38]. In another series of eight patients with la-cSCC treated with cetuximab and radiotherapy, more durable disease control was reported, with an estimated probability of PFS at 24 months of 83.3% [35].

Neoadjuvant approach could overcome the problem of short-lasting responses. In neoadjuvant setting, cetuximab was used in monotherapy or combined with cisplatin or carboplatin and 5-FU in a group of 34 patients suffering from unresectable cSCC. These protocols showed good response and are very promising, since 92% of the cetuximab plus chemotherapy group and 55% of the monotherapy group became eligible for surgery after 9 weeks [36]. An ongoing study is investigating cetuximab as a potential neoadjuvant treatment in la-SCC (NCT02324608).

##### Standard dosage

Initial dose of 400 mg/m^2^, followed by subsequent weekly doses of 250 mg/m^2^, intravenous administration.

##### Contraindications

Severe hypersensitivity to cetuximab; thorough risk/benefit assessment is necessary in case of pregnancy, lactation or inadequate pregnancy prevention.

##### Main drug interactions

If associated to platinum-based chemotherapy, cetuximab increases the frequency of severe leucopenia, neutropenia, and infectious complications. When administered together with fluoropyrimidines, cetuximab increases the risk of cardiac ischemia and palmar-plantar erythrodysesthesia. Association with capecitabine and oxaliplatin increases the frequency of severe diarrhea [39].

##### Main side effects

Common adverse reactions include skin rashes (>80% of treated patients) such as acneiform rash, pruritus, dry skin, hypertrichosis and nail alterations, *S. aureus* skin infections, hypomagnesemia (>10%), mild to moderate infusion reactions (>10%) or severe infusion reactions (<1%), headache, conjunctivitis, diarrhea, nausea, vomiting, anorexia, mucositis, increase in liver enzymes levels, fatigue, and hypocalcemia. More severe but quite rare adverse effects are deep venous thrombosis, pulmonary embolism, pulmonary interstitial disease, and Stevens-Johnson syndrome [37, 39, 40].

##### Cost/cost-effectiveness

The cost is elevated.

#### Panitumumab

In an open-label phase II trial involving 16 patients with la-cSCC, panitumumab was characterized by a best RR of 31%, DCR at 6 weeks of 69%, and median PFS of 8 months, showing comparable efficacy to cetuximab [16]. Another recent retrospective study involved 25 individuals with unresectable cSCC, treated with panitumumab monotherapy or with a combination of this drug plus radiotherapy: the best RR, DCR at 6 weeks, and median PFS of the whole group were 52%, 84%, and 6.9 months, respectively [41]. Notably, all patients with complete response and more than 50% of patients with partial response had received concurrent radiotherapy [41].

##### Standard dosage

6 mg/kg every 2 weeks, intravenous administration.

##### Contraindications

Hypersensitivity to active principle or bulking agents and severe adverse reactions to panitumumab, pulmonary fibrosis, or interstitial pneumonia. Thorough risk/benefit assessment is necessary in case of pregnancy, lactation, or inadequate pregnancy prevention.

##### Main drug interactions

Avoid association with 5-fluorouracil, leucovorin, and irinotecan (increased incidence of severe diarrhea); avoid association with bevacizumab and chemotherapy (increased incidence of toxicity and mortality) [39].

##### Main adverse effects

Cutaneous rashes represent the most common drug-related toxicity, arising in 94% of treated patients (severe reactions: 23%; life-threatening reactions: <1%), sometimes with relevant infectious complications such as cellulitis, necrotizing fasciitis, and sepsis. Other adverse effects include nail changes, dyspnea, cough, development or worsening of preexistent interstitial lung disease, hypomagnesemia and hypocalcemia, hypokalemia, nausea, diarrhea, decreased appetite, acute renal failure (as consequence of severe diarrhea and dehydration), conjunctivitis, keratitis, abdominal and dorsal pain, fatigue, and insomnia [16, 39, 41].

##### Cost/cost-effectiveness

The cost is high.

#### EGFR tyrosine kinase inhibitors

Gefitinib, erlotinib, lapatinib, and dacomitinib are EGFR tyrosine kinase inhibitors used in non-small-cell lung cancer and other malignancies such as pancreatic and breast cancer. Although not approved for NMSC, their use has been reported for cSCC not eligible for surgery or radiotherapy [17–20].

In non-randomized phase II studies involving patients with la-cSCC, gefitinib (dose: 250 mg orally daily) was characterized by overall RR of 16%, DCR at 8 weeks of 51%, and a 3.8 months PFS [18], while erlotinib (dose: 150 mg orally daily) showed an overall RR of 10%, DCR of 72% at 6 weeks, and a median PFS of 4.7 months [17]. Gefitinib study involved 37 evaluable patients, while erlotinib study included 29 individuals; in both cases, only partial responses were observed (no complete responses were registered) [17, 18].

In an open-label prospective study, lapatinib (1500 mg/day for 56 days) used in neoadjuvant setting reduced cSCC size in two of eight patients, together with a significant decrease of concomitant actinic keratoses [19]. In another open-label, multicentric, uncontrolled phase II trial involving patients with unresectable or metastatic cSCC, dacomitinib was administered. Considering the 42 evaluable patients, overall RR was 28% (2% complete response, represented by only one patient; 26% partial response), DCR was 86%, and median PFS was 6 months [20].

##### Standard dosage

Oral administration, see text above for single drugs

##### Contraindications

Hypersensitivity to active principle or bulking agents, history of solid organ transplant, pregnancy, and lactation. Patients with impaired hepatic function and patients with a slow metabolizer CYP2D6 genotype should be strictly monitored for adverse effects.

##### Main drug interactions

CYP3A4 inhibitors (i.e., ritonavir, saquinavir, telithromycin, ketoconazole, itraconazole, voriconazole, posaconazole, nefazodone) increase EGFR tyrosine kinase inhibitor concentration and CYP3A4 inductors (i.e., rifampicin, rifabutin carbamazepine, phenytoin, phenobarbital, and St. John’s herb or *Hypericum perforatum*) reduce it. In patients using warfarin, international normalized ratio (INR) should be monitored, because there is an increased risk of its elevation and hemorrhagic events. Gastric pH elevation (such as in proton pump inhibitors use) can reduce drug absorption [42, 43].

##### Main adverse effects

Common adverse effects for this drug class are acneiform skin rashes, pruritus, nail changes, fatigue, nausea, vomiting, diarrhea, anorexia, conjunctivitis, hemorrhage, and epistaxis, increase of liver enzymes, and creatinine levels. Interstitial pulmonary disease is rarer but can be severe. Lapatinib has also been associated with left ventricular ejection fraction reduction [17–20, 42].

##### Cost/cost-effectiveness

The cost is elevated.

## Pharmacologic treatments: immunotherapy

Immunotherapy has become a cornerstone for advanced and metastatic cutaneous tumor management and is currently indicated for melanoma, cSCC, and MCC [44••]. This treatment acts by inhibiting immune checkpoints, such as cytotoxic T-lymphocyte-associated protein 4 (CTLA-4), programmed cell death receptor-1 (PD-1), and programmed cell death ligand-1 (PD-L1), eventually improving the activity of the immune system against the tumoral cells and reducing regulatory T cell-mediated immunosuppression [44••]. This mechanism is very convenient if the targeted tumor has an important mutational burden leading to antigenicity, such as melanoma and NMSC [45].

### Anti-programmed cell death receptor-1 immune checkpoint inhibitor

#### Cemiplimab

##### Cutaneous squamous cell carcinoma

Cemiplimab (a human monoclonal anti-PD-1 antibody) is indicated for m-cSCC and la-SCC in patients who are not eligible for curative surgery or radiotherapy. The pivotal trial that led to drug approval was a phase I/II study (EMPOWER-CSCC-1) including a phase I cohort with la-cSCC and m-cSCC (26 patients) and phase II cohort with m-cSCC (59 patients) [46]. For the phase I cohort, RR was 50% (95% CI, 30 to 70) and DCR was 65% (95% CI, 44 to 83). The duration of response exceeded 6 months in more than half of responders [46]. For the phase II cohort, RR was 47% (95% CI, 34 to 61) and DCR was 61% (95% CI, 47 to 74). The estimated probability of PFS at 12 months was 53% (95% CI, 37 to 66) and the estimated probability of overall survival at 12 months was 81% (95% CI, 68 to 89) [46].

These good results underline that cemiplimab could be also useful in neoadjuvant setting for cSCC: currently, several ongoing trials are investigating this hypothesis (NCT04428671, NCT03565783). Interestingly, one of them adopts intralesional cemiplimab administration (NCT03889912).

##### Basal cell carcinoma

According to a case report, a patient with recurrent metastatic BCC was treated with cemiplimab, obtaining partial response and a PFS of 32 weeks [47]. Cemiplimab is also being used in an ongoing study involving la-BCC resistant to therapy with sonic hedgehog pathway inhibitors (NCT03132636).

##### Standard dosage

350 mg every 3 weeks, intravenous administration.

##### Contraindications

Hypersensitivity to active principle or bulking agents, pregnancy, and lactation. Moreover, exclusion criteria from the studies were autoimmune disease, concurrent cancer, history of solid organ transplant, prior treatment with anti-PD-1/PD-L1 blocking antibodies or other immune checkpoint inhibitor therapy, infection with HIV, hepatitis B or hepatitis C [46].

##### Main drug interactions

Avoid systemic steroids or immunosuppressants before cemiplimab administration (potential reduction of cemiplimab efficacy).

##### Main adverse effects

The most common adverse reactions are fatigue (29%), skin rash (25%), diarrhea (22%), nausea (19%), musculoskeletal pain (17%), pruritus (15%), constipation (12%), decreased appetite (10%), liver enzymes, and creatinine serum level elevation, lymphopenia, and anemia. Infusion reactions are generally not severe, whereas immune-related adverse effects can be irreversible, serious, and rarely fatal. Among them, immune-related pneumonitis (2.4%), hepatitis (2.1%), colitis (0.9%), nephritis with renal dysfunction (0.6%), and endocrinopathies such as thyroiditis, hypophysitis, adrenal insufficiency, and diabetes mellitus type 1 [46, 48].

##### Special points

Cemiplimab is the only drug approved for systemic treatment in cSCC [44••].

##### Cost/cost-effectiveness

The cost is elevated.

#### Pembrolizumab and nivolumab

Pembrolizumab and nivolumab are human monoclonal anti-PD-1 antibodies approved for advanced melanoma and other malignancies including metastatic non-small cell lung cancer, Hodgkin lymphoma, urothelial cancer, renal cancer, head and neck SCC, and Merkel cell carcinoma [49].

##### Cutaneous squamous cell carcinoma

Pembrolizumab and nivolumab use for unresectable or metastatic cSCC was initially described in case series [49–54]. Good results were reported in an open-label, single-arm, phase II study (KEYNOTE-629) involving 105 patients with recurrent or metastatic cSCC treated with pembrolizumab: RR was 34.3% (95% CI, 25.3% to 44.2%; with 4 complete responses, 32 partial responses), DCR was 52.4% (95% CI, 42.4% to 62.2%), and median PFS was 6.9 months (95% CI, 3.1 to 8.5 months) [55]. Comparable results were described in an interim analysis of the phase 2 CARSKIN trial (NCT02883556), regarding patients with unresectable cSCC treated with pembrolizumab [56].

Moreover, there are currently ongoing studies evaluating novel therapeutic approaches in la-cSCC, such as pembrolizumab monotherapy (NCT02964559), nivolumab monotherapy (NCT03834233), pembrolizumab associated to cetuximab (NCT03082534).

##### Basal cell carcinoma

Regarding la-BCC and m-BCC, there are case reports of pembrolizumab or nivolumab use, with variable results [54] and also an ongoing study evaluating nivolumab monotherapy or in combination with ipilimumab (NCT03521830). In a non-randomized open label study involving 16 patients with la-BCC, either pembrolizumab monotherapy or pembrolizumab plus vismodegib were administered. For the first group, RR at 18 weeks was 44% (4/9 patients; 95% confidence interval 14–79%), while for the combination therapy group, RR was 29% (2/7 patients; 95% confidence interval 4–71%) [57].

An ongoing study is investigating neoadjuvant pembrolizumab use in advanced BCC (NCT04323202).

##### Merkel cell carcinoma

As far as concerns advanced Merkel cell carcinoma, durable tumor regression has been recently obtained in patients treated with pembrolizumab in the KEYNOTE 017 trial (objective RR of 56% and median PFS of 16.8 months) and United States Food and Drug Administration (FDA) approved this drug as first-line therapy for MCC [58].

In the phase I/II CheckMate 358 study, nivolumab was used as neoadjuvant option in MCC, before surgical excision [59]. Among 36 patients who underwent surgery, 17 (47.2%) achieved a pathologic complete response and none of them showed relapse during observation [59].

##### Standard dosage

The most common therapeutic scheme for pembrolizumab is 200 mg every 3 weeks, while for nivolumab is 240 mg every 2 weeks, intravenous administration.

##### Contraindications

Hypersensitivity to active principle or bulking agents, history of solid organ transplant, autoimmune disease, pregnancy, and lactation.

##### Main drug interactions

Avoid systemic steroids or immunosuppressants before administration (potential reduction of efficacy).

##### Main adverse effects

Nonspecific adverse effects such as fatigue, nausea, diarrhea, skin rashes, and pruritus are common and reported in 10–30% of cases. Infusion reactions are common but mostly mild. Other notable adverse effects are liver enzymes and creatinine serum level elevation, musculoskeletal pain, arthralgia, dyspnea and cough, hypertension, xerostomia and xerophthalmia, headache, insomnia, anemia, thrombocytopenia, leukopenia, alteration of ion serum levels. Immune-related toxicity can be irreversible, severe, and rarely fatal: immune-related pneumonitis, nephritis, colitis, hepatitis, and endocrinopathies such as thyroiditis hypophysitis, adrenal insufficiency, and diabetes mellitus are reported [60,61].

##### Cost/cost-effectiveness

The cost is elevated.

### Anti-cytotoxic T-lymphocyte-associated protein 4 immune checkpoint inhibition

#### Ipilimumab

Cytotoxic T-lymphocyte-associated protein 4 (CTLA4) is a T cell protein receptor that prevents T cell activation when bound to a costimulatory protein receptor called B7, localized on the surface of an antigen-presenting cell. Ipilimumab is an anti-CTLA4 monoclonal antibody (IgG1k), approved for melanoma and renal cell cancer (in monotherapy or in association with nivolumab).

##### Cutaneous squamous cell carcinoma

Data regarding ipilimumab use in NMSC are limited to case reports. A patient with metastatic melanoma and concurrent metastatic cSCC had a durable remission of both malignancies [62].

##### Basal cell carcinoma

Regression of an advanced BCC was similarly reported in a patient exposed to ipilimumab for a concurrent metastatic melanoma [63]. There is an ongoing study involving la-BCC or m-BCC which evaluates ipilimumab in association with nivolumab in one of the arms (NCT03521830).

##### Standard dosage

3 mg/kg intravenously every 3 weeks, for 4 doses.

##### Contraindications

Hypersensitivity to active principle or bulking agents, history of solid organ transplant, autoimmune disease, pregnancy, and lactation.

##### Main drug interactions

Avoid systemic steroids or immunosuppressants before administration (potential reduction of ipilimumab efficacy). Strict monitoring of patients using anticoagulant therapy is advisable (increased risk of gastrointestinal hemorrhage).

##### Main adverse effects

Skin rashes, pruritus, dermatitis, vitiligo, musculoskeletal pain, arthralgia; anorexia, diarrhea, nausea, gastrointestinal bleeding, impaired liver function; anemia, lymphopenia, dehydration, hypokalemia, headache, peripheral neuropathy, blurred vision, hypotension, dyspnea, and cough. Other immune-related adverse effects can be irreversible, severe, and rarely fatal but are not so common: among them, pneumonitis, nephritis, hepatitis, and endocrinopathies such as thyroiditis, hypophysitis, and adrenal insufficiency are reported [60, 61].

##### Cost/cost-effectiveness

The cost is elevated.

## Pharmacologic treatments: systemic chemotherapy

Currently, there is no approved systemic chemotherapy agent for locally advanced and metastatic NMSC, but platinum-based protocols (cisplatin or carboplatin) have been sometimes adopted, especially for metastatic cSCC [64–66]. Therapeutic attempts with bleomycin, methotrexate, adriamycin, gemcitabine, doxorubicin, paclitaxel, ifosfamide, cyclophosphamide, etoposide, and antimetabolite pyrimidine analogs (such as 5-fluorouracil and capecitabine) are also reported [67••, 68].

According to observational studies involving advanced and metastatic NMSC, overall RR is higher for polychemotherapy rather than monotherapy, but the latter is generally more tolerated and associated to less severe toxicity (essential factor for elderly people or patients with comorbidities) [67••, 69, 70].

### Cutaneous squamous cell carcinoma

A retrospective study involving a group of 25 patients with la-SCC and m-SCC treated with systemic therapies (chemotherapy regimens in large part) showed a RR of 44% [69]. Unfortunately, only partial responses were reported and recurrence was the norm within months (median PFS of 5.5 months) [69]. Of note, cetuximab was included among systemic therapies in this analysis [69]. Comparable results were reported in a recent meta-analysis involving 60 cases of m-SCC treated with cisplatin: overall RR was 45% (complete response 22%; partial response 25%) and median disease-free survival in responders was 14.6 months [64].

In neoadjuvant setting, cisplatin, bleomycin, and 5-fluorouracil led to good results in some dated case series, eventually allowing local control with radiation and/or surgery with better functional and cosmetic results [71–73].

#### Basal cell carcinoma

Systemic platinum-based chemotherapy was also used in la-BCC and m-BCC, achieving overall RR of up to 77–83%, with complete response in up to 45% of treated lesions, although without long-term remissions (relapse in months) [65, 66].

In neoadjuvant setting, cisplatin was used prior to surgery in combination with doxorubicin, adriamycin ± cyclophosphamide, or bleomycin, showing some efficacy, but the evidence is not so strong (small-size and outdated case series) [70].

#### Merkel cell carcinoma

In MCC, neoadjuvant cisplatin, etoposide, and cyclophosphamide regimen led to local control in two patients, allowing curative surgery associated with adjuvant radiation therapy [68].

### Cisplatin

Platinum-based protocols represent the most common approach, if chemotherapy is chosen.

#### Standard dosage

Dosage of intravenous cisplatin varied from 20 to 100 mg/m^2^ in different studies (depending on mono or polychemotherapy) [69, 70].

#### Contraindications

Hypersensitivity to active principle or bulking agents, pregnancy and lactation, renal dysfunction, myelosuppression, auditory alteration, and platinum-related neuropathy.

#### Main drug interactions

Avoid concomitant nephrotoxic agents (such as aminoglycosides, amphotericin B, contrast medium) or ototoxic drugs (for example, aminoglycosides and loop diuretics). Avoid live attenuated vaccines (risk of fatal systemic disease). Strict patient monitoring is advisable in case of concomitant oral anticoagulant use. Contemporary use of antihistamines can mask ototoxicity symptoms. Exposition to anticonvulsant drug may decrease [58–61, 63, 64].

#### Main adverse effects

Hematologic (leukopenia, thrombocytopenia, anemia) and gastrointestinal adverse effects (anorexia, nausea, vomit, diarrhea), auditory disturbances, renal alterations (renal failure, nephrotoxicity, hyperuricemia), arrythmias [64–66, 67••, 69, 70].

#### Cost/cost-effectiveness

This treatment is less expensive than targeted therapy or immunotherapy; multiple infusions are required.

## Interventional procedures

### Intralesional chemotherapy

Intralesional administration of chemotherapy agents such as 5-fluorouracil, bleomycin, and interferon has been used in BCCs, SCCs, and keratoacanthomas [74]. 5-Fluorouracil is an antimetabolite and structural analog of uracil and acts by disrupting DNA and RNA [75], bleomycin is cytotoxic and causes DNA damage, while interferon acts as an antiproliferative and immune-stimulating drug [74]. Major contraindications for intralesional chemotherapy are pregnancy and lactation, while caution is advisable in patients with renal and hepatic disease [74]. Only local discomfort is generally reported (pain, erythema, crusting, ulceration, necrosis), but systemic side effects are possible (such as cytopenia, gastrointestinal upset, hepato- and nephrotoxicity, teratogenesis) [74].

#### Intralesional methotrexate

Methotrexate is a folic acid analog, which inhibits DNA synthesis in actively dividing cells, irreversibly binding to dihydrofolate reductase and therefore blocking the formation of tetrahydrofolate [76]. This mechanism prevents the downstream synthesis of the nucleotide thymidine [76].

Intralesional methotrexate (MTX) showed efficacy for primary cSCC, in particular for well-differentiated subtype and keratoacanthomas [76–79]. Its use as neoadjuvant treatment was investigated in a retrospective study on 86 cSCCs [77]. The group undergoing neoadjuvant intralesional MTX administration before surgery (half of the lesions, *n*=43) showed an average reduction in cSCC size of 0.52 cm^2^, whereas the group scheduled for excision was characterized by 0.49 cm^2^ area increase [77]. The data of this study also suggested a reduced rate of complex surgical reconstructions (local flaps or grafts) in treated tumors, especially in lesions ≥2 cm in diameter [77].

##### Standard procedure

No standard procedure is established. For example, in the aforementioned study, MTX was administered into the base of the tumor until it acquired a yellowish color, with a mean MTX quantity of 0.74 mL (0.1–1.3 mL), corresponding to 2.5–32.5 mg [77]. One or more injections (repeated weekly) are usually performed [76–79]. A dose of 10 mg of folic acid is sometimes administered 24 h after the procedure [79].

##### Contraindications

Hypersensitivity to methotrexate, pregnancy, or lactation. Hematological abnormalities, liver or kidney disease may be considered relative contraindications [77].

##### Adverse effects and complications

Pain, erythema, crusting, ulceration, and necrosis are quite common [78]. Topical anesthesia or concomitant use of lidocaine and epinephrine may help reduce the pain; moreover, epinephrine-induced vasoconstriction decreases diffusion of the chemotherapy agent [78]. Pancytopenia occurring after a 25 mg intralesional MTX injection has been previously reported in patients with hemodialysis-dependent renal failure [76]. Although these adverse events are rare, performing a baseline and post-injection complete blood cell count is advisable to detect for potential subclinical MTX-induced cytopenia [76].

##### Cost/cost-effectiveness

This technique is relatively inexpensive.

### Electrochemotherapy

As far as concerns electrochemotherapy (ECT), this procedure uses electrical pulses to increase absorption of a chemotherapy agent (usually bleomycin, less frequently cisplatin) administered intravenously or intratumorally [80].

ECT is used for primary, locally advanced and metastatic NMSC and is considered a safe and well-tolerated treatment. In a multicenter prospective study involving a total of 105 patients affected by primary or recurrent skin cancer of the head and neck area, ECT reached an objective RR of 97% for BCC and 79% for cSCC after 2 months [81]. Of note, the best response was obtained in tumors smaller than 3 cm in diameter [81].

#### Standard procedure

Bleomycin is administrated either intravenously or intratumorally, depending on the site, size, number of skin nodules, and comorbidity. The procedure is performed under general or local anesthesia: the first option is preferred if numerous or large nodules are treated. Electrical pulses (eight pulses of 100 ms duration, amplitude of 1000–1300 V/cm) are delivered with an electroporator immediately after the intratumoral injection or 8 min after the intravenous injection of bleomycin. Treated lesions form an eschar as necrosis occurs about 10–14 days after the procedure [80–82]**.**

#### Contraindications

Pregnancy and lactation, history of hypersensitivity to bleomycin or cisplatin. In presence of a history of pulmonary disease or renal impairment, a dose reduction may be required [80].

#### Complications

Pain at the electroporation site is quite common but can be normally managed with paracetamol or other simple analgesic drugs. Acute toxicity is minimal; chemotherapy adverse effects are rare (bleomycin pulmonary fibrosis is reported, but it is exceptional) [80].

#### Cost/cost-effectiveness

An equipped operating room and specialized professionals are required, increasing the costs. General anesthesia is sometimes necessary.

### Photodynamic therapy

Photodynamic therapy (PDT) uses 5-aminolevulinic acid (5-ALA) or methylaminolevulinic acid (MAL). They both are precursors of heme in its biosynthetic pathway and form intermediate porphyrins such as the photoactive compound protoporphyrin IX. When excited by light of an adequate wavelength, protoporphyrin IX forms reactive oxygen species, leading to tumoral cell death by necrosis and apoptosis (the mechanisms are direct cytotoxicity, damage to the tumor vasculature, and activation of the immune response) [83].

Actinic keratoses, superficial BCC, and Bowen’s disease/squamous cell carcinoma in situ are approved indications for PDT [84]. This technique can be also used for nodular BCCs, but response is inferior and recurrence rate is high, especially for lesions thicker than 2–3 mm; in addition, heavily pigmented lesions such as pigmented BCCs show less response [84, 85].

PDT was used as neoadjuvant treatment in reducing lesion size for facial tumors (BCCs and SCCs) localized in functionally and aesthetically sensitive areas, showing improvement of the lesions and area reduction for most lesions [85, 86]**.**

#### Standard procedure

After mild curettage of target lesion, ALA preparation or MAL ointment is applied. An incubation time is necessary (usually 3–6 h) and treated areas are subsequently exposed to light, generally using lamps [83,84].

#### Contraindications

Known history of porphyria or allergic reactions to active ingredients or bulking agents of the applied sensitizers [83].

#### Adverse effects and complications

Stinging pain and burning sensation during light exposure is quite common. Subsequent localized erythema and edema in the treated area are usually seen. Over the days following the treatment, erosions and crusting precede reepithelization [83].

#### Cost/cost-effectiveness

In addition to the photosensitizer cost, the procedure itself requires special equipment and qualified personnel. More than one session is usually needed (at least two or three treatments) [85, 86]**.**

## Emerging therapies

Considering the high mutational burden and antigenicity of NMSC, immunotherapy appears to be one of the most promising fields. Anti-PD-L1 antibodies such as avelumab, atezolizumab, and cosibelimab act by interfering with the PD-1 pathway, similarly to pembrolizumab and nivolumab.

Of note, avelumab has been approved by FDA for metastatic MCC and has also been used for this malignancy in neoadjuvant setting: of ten eligible patients that underwent surgery, four (40%) reached a pathological complete response [87•].

Ongoing studies for advanced cSCC are evaluating avelumab with or without cetuximab (NCT03944941), avelumab with radiotherapy (NCT03737721), atezolizumab together with cobimetinib, MEK inhibitor (NCT03108131), cosibelimab (NCT03212404), and SL-279252, a PD-1-FC-OX40L checkpoint fusion protein that acts as an immune system agonist (NCT03894618).

Moreover, intralesional administration of talimogene laherparepvec (T-VEC), a genetically modified herpes simplex virus-1, is currently being evaluated for advanced NMSC (NCT03458117).

## Conclusion and summary

Given the actual evidence, targeted therapy with sonic hedgehog inhibitors for BCC and immunotherapy for all NMSCs (in particular, cSCC and MCC) are the most promising strategies in a neoadjuvant setting, being much more selective than traditional chemotherapy. On the other hand, targeted therapy for cSCC appears to be at its dawn, given the modest results gained by EGFR inhibitors. A more detailed comprehension of squamous cell carcinogenesis is needed to develop new strategies. Unfortunately, all these aforementioned novel treatments are actually quite expensive and immunotherapy can lead to severe and irreversible adverse effects. In addition, not all treated lesions show good response and recurrence rates are elevated; therefore, further research is required to overcome these difficulties. Finally, we do not have to forget that also other less expensive approaches exist, such as intralesional chemotherapy, PDT, and electrochemotherapy. Neoadjuvant approach for NMSC is a promising field and will be probably approved in the near future.
